# Dynamic Contrast-Enhanced MRI Kinetic Curve-Driven Parametric Radiomics for Predicting Breast Cancer Molecular Subtypes: A Multicenter and Interpretable Study

**DOI:** 10.3390/tomography12020027

**Published:** 2026-02-22

**Authors:** Ting Wang, Jing Gong, Simin Wang, Shiyun Sun, Jiayin Zhou, Luyi Lin, Dandan Zhang, Chao You, Yajia Gu

**Affiliations:** 1 Department of Radiology, Fudan University Shanghai Cancer Center, Shanghai 200032, China; wtfighting418@126.com (T.W.); gongjing1990@163.com (J.G.);; 2 Department of Oncology, Shanghai Medical College, Fudan University, Shanghai 200032, China; 3 Department of Radiology, Renji Hospital, School of Medicine, Shanghai Jiao Tong University, Shanghai 200127, China

**Keywords:** breast cancer, dynamic contrast-enhanced magnetic resonance imaging, time-intensity curve, radiomics

## Abstract

**Background/Objectives**: To investigate and develop a non-invasive parametric radiomics model derived from dynamic contrast-enhanced MRI (DCE-MRI) time-intensity curve (TIC) kinetics for predicting breast cancer molecular subtypes (HR+/HER2−, HER2+ and triple-negative breast cancer). **Methods**: This multicenter retrospective study enrolled 935 female patients with histologically confirmed breast cancer who underwent pretreatment breast DCE-MRI from August 2017 to July 2022. Based on the wash-in rate (WIR) and the area under the TIC, the original multiphase DCE-MRI images were converted into two types of parametric images. Radiomics features were extracted from TIC-WIR and TIC-Area images and analyzed using low variance filtering, the elimination of highly correlated features, and the least absolute shrinkage and selection operator regression. The categorical boosting algorithm was employed to develop multiclass prediction models for breast cancer molecular subtyping. A TIC-Combined model was further established by integrating the calibrated probability outputs of the TIC-WIR and TIC-Area models using a decision-level fusion strategy. The discrimination, calibration, and interpretability of the models were evaluated in the study datasets. **Results**: The TIC-Combined model achieved superior predictive performance in both the internal validation set (micro-average AUC: 0.79, macro-average AUC: 0.77) and the external validation set (micro-average AUC: 0.77, macro-average AUC: 0.75). For subtype-specific classification by the TIC-Combined model, the highest one-vs-rest AUCs were 0.81 for triple-negative breast cancer in the internal validation set and 0.76 for HER2+ breast cancer in the external validation set. The TIC-Combined model also showed good calibration and high interpretability which ensured reliable predictions and provided clear insights into feature importance. **Conclusions**: Interpretable parametric radiomics from TIC-derived parametric maps links kinetic features to molecular phenotypes, enabling accurate and non-invasive classification of breast cancer molecular subtypes.

## Simple Summary

Reliable prediction of breast cancer molecular subtypes is critical for guiding personalized treatment and improving clinical outcomes. Our study proposes an innovative, non-invasive parametric radiomics approach derived from DCE-MRI time-intensity curve kinetics. By converting original multiphase images into parametric images, and applying advanced radiomics and machine learning methods, we developed and validated interpretable models capable of accurately classifying breast cancer molecular subtypes. The findings in this study highlight the potential of DCE-MRI kinetic-driven radiomics to provide clinically meaningful, non-invasive subtype prediction, thereby supporting precision oncology.

## 1. Introduction

Breast cancer is the most common diagnosed malignancy and the leading cause of cancer-related deaths in women worldwide [1]. It is a heterogeneous disease with highly variable prognosis for the differences in the genomic, transcriptomic, and proteomic characteristics of the cancer cells [2]. Breast tumors are clinically categorized into three treatment-oriented subtypes according to the expression of the estrogen receptor (ER), progesterone receptor (PR), and human epithelial growth factor receptor 2 (HER2). Patients with hormone receptor-positive/HER2-negative (HR+/HER2-) cancer are eligible for endocrine therapy [3]. And HER2-targeted therapy is the current standard of care for HER2-positive (HER2+) breast cancer [4]. While triple-negative breast cancer (TNBC) does not have specific target receptors and chemotherapy remains the reference treatment [5].

Radiomics has emerged as a quantitative imaging analysis framework that converts medical images into high-dimensional, mineable data, enabling the extraction of features that characterize tumor intensity, texture, shape, and spatial heterogeneity. By capturing imaging patterns that are not readily appreciable by visual inspection, radiomics provides a non-invasive approach to probing intratumoral heterogeneity, which is increasingly recognized as a key imaging surrogate of underlying tumor biology. Radiomics has been increasingly recognized as a valuable imaging biomarker, with growing evidence supporting its integration with multimodal data for precision oncology applications [6,7]. In breast imaging, radiomics has shown promising potential for tumor characterization, molecular subtype prediction, and treatment response assessment.

Dynamic contrast-enhanced magnetic resonance imaging (DCE-MRI) enables non-invasive assessment of tissue hemodynamic characteristics, offering insights into perfusion and vascular permeability. Breast cancer molecular subtypes exhibit distinct tumor micro-environment patterns that reflect underlying biological heterogeneity [8]. Among the components of the tumor micro-environment, tumor angiogenesis is a key determinant of sustaining tumor cell survival and contributes significantly to their growth, invasion, and metastasis [9]. The formation of an abnormal vascular network characterized by disorganized and immature blood vessels leads to impaired perfusion and increased vascular permeability [10], resulting in significant alterations in tumor hemodynamics that can be captured by DCE-MRI.

The time-intensity curve (TIC) derived from DCE-MRI provides valuable kinetic information and has been applied in breast tumor classification and treatment response prediction [11,12]. However, conventional TIC analysis typically relies on empirical parameters extracted from manually delineated regions of interest, which are highly influenced by inter-observer variability and averaging effects that limit the ability to capture the spatial heterogeneity of contrast enhancement within the tumor. In contrast, parametric imaging preserves spatial resolution by generating voxel-wise parametric maps that can retain the spatial distribution of contrast uptake patterns throughout the entire tumor volume. Motivated by the complementary strengths of conventional TIC-based features characterizing the kinetic behaviors of tumor tissue over time and parametric imaging revealing localized differences, we sought to integrate spatiotemporal information for a more comprehensive characterization of tumor enhancement heterogeneity.

In this study, a new approach generating voxel-wise parametric maps based on TIC is proposed, bridging the gap between spatial and temporal dimensions in DCE-MRI images. Further, the resulting voxel-wise parametric images allow for the quantification of intratumoral heterogeneity through radiomics analysis with the aim of assessing the effectiveness in predicting breast cancer molecular subtypes.

## 2. Materials and Methods

### 2.1. Study Datasets

**Figure 1:**
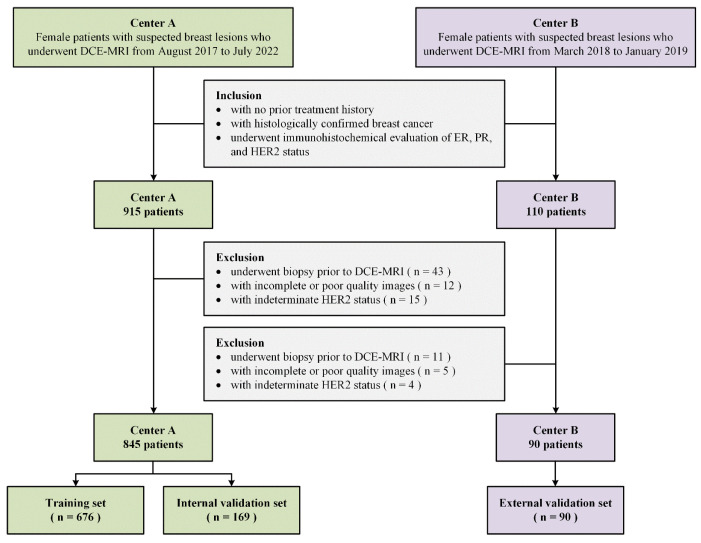
Flowchart of participant selection.

This multicenter retrospective study was approved by the local institutional review boards of Center A (Fudan University Shanghai Cancer Center, FUSCC; approval number: 2004216-14) and Center B (Renji Hospital Affiliated to Shanghai Jiao Tong University School of Medicine, RH; approval number: SK2020-002) with a waiver of informed consent. Female patients presenting with suspected breast lesions who underwent baseline DCE-MRI were collected from August 2017 to July 2022 at FUSCC and from March 2018 to January 2019 at RH. Data from the patients who met the following criteria were included: (1) with no history of treatment prior to DCE-MRI; (2) histologically diagnosed with breast cancer; and (3) ER, PR, and HER2 expression status were available. Additionally, the exclusion criteria were as follows: (1) biopsy was performed prior to DCE-MRI; (2) DCE-MRI scans with incomplete data or poor-quality images; and (3) HER2 status was indeterminate. The overall patient selection process is summarized in Figure 1.

### 2.2. Histopathological Analysis

The expression of ER, PR, and HER2 were determined by IHC analysis through available biopsy samples or resected surgical specimens. The ER and PR statuses were considered positive if at least 1% of tumor cells exhibited positive nuclear staining. According to the updated 2023 American Society of Clinical Oncology/College of American Pathologists guideline on HER2 testing in breast cancer [13], HER2 positivity was defined as an IHC score of 3+ or an IHC score of 2+ with a positive FISH result; otherwise, it was defined as HER2 negative. Accordingly, breast cancer molecular subtypes were classified into three subtypes including HR+/HER2− (ER-positive and/or PR-positive and HER2-negative), HER2+ (HER2-positive regardless of ER and PR status), and TNBC (ER-, PR-, and HER2-negative).

### 2.3. MRI Protocols and Image Registration

Baseline breast MRI scans were performed on 3T scanners (FUSCC: MAGNETOM Skyra, Siemens Healthcare, Erlangen, Germany; RH: Achieva, Philips Healthcare, Best, The Netherlands) employing dedicated breast coils with patients in the prone position. Axial DCE-MRI acquisition was conducted using a fat-suppressed three-dimensional T1-weighted fast spoiled gradient echo sequence before and after intravenous injection of gadolinium-based contrast agents (gadopentetate dimeglumine, 0.1 mmol/kg body weight, at a rate of 2 mL/s; Magnevist; Bayer HealthCare Pharmaceuticals, Berlin, Germany), followed by a 20 mL saline flush. Detailed imaging parameters are listed in Table 1.

To correct for patient motion, post-contrast images were aligned to the pre-contrast image using a registration algorithm that combined rigid and non-rigid B-splines transforms in a multi-resolution scheme [14]. Multiphase DCE-MRI registration was implemented using the Elastix tool. Specifically, the parameter configuration provided in the Elastix Model Zoo (Par0032) was adopted. The corresponding parameter files are publicly available in the model directory of the Elastix Model Zoo repository (https://github.com/SuperElastix/ElastixModelZoo/tree/master/models/Par0032, accessed on 29 April 2025). All subsequent image processing and analysis were conducted on the registered images.

### 2.4. TIC-Based Parametric Image

Initially, the signal intensities of all post-contrast phases were normalized by dividing by the pre-contrast signal intensity. Then, the wash-in rate (WIR) and the area under the TIC were generated for each voxel. Specifically, wash-in rate was defined as (I_peak_norm_ − 1)/T_peak_, where I_peak_norm_ indicates the normalized peak signal intensity within the first three post-contrast phases, and T_peak_ is the time in seconds relative to contrast injection at which the corresponding peak intensity occurs. Meanwhile, the area under the TIC was calculated by using the trapezoidal rule, integrating between the time of contrast agent administration and the time of the last post-contrast phase, and subsequently normalized by the total acquisition time. Ultimately, two derived images were generated by computing these two TIC-based parameters on a voxel-wise basis from the original multiphase DCE-MRI images, referred to as the TIC-WIR and TIC-Area images.

### 2.5. Tumor Segmentation and Parametric Radiomics Feature Extraction

The tumors were manually delineated by a senior radiologist with ten years of experience in breast imaging via ITK-SNAP software (http://www.itksnap.org/, version 3.8.0) based on the first post-contrast phase image. The final tumor annotations were reviewed and confirmed by another senior radiologist who had specialized in breast imaging for more than two decades. All tumor segmentations by the two radiologists were performed blinded to the breast cancer molecular subtype labels.

To ensure consistency and comparability in parametric radiomics analysis across the images [15], voxel size was resampled to 1 mm × 1 mm × 1 mm resolution using the B-spline curve interpolation algorithm, and gray-level discretization with a fixed bin width of 5 was used. For each derived image and the peak enhancement phase image of the tumor, a total of 851 radiomics features per patient were extracted from the original and wavelet-transformed images using Python PyRadiomics (version 3.0.1) [16], including first-order intensity features, shape features, and texture features (gray level co-occurrence matrix [GLCM], gray level size zone matrix [GLSZM], gray level run length matrix [GLRLM], neighboring gray tone difference matrix [NGTDM], and gray level dependence matrix [GLDM]).

### 2.6. Parametric Radiomics Feature Selection and Predictive Model Construction

A three-step feature selection approach for high-dimensional parametric radiomics data was performed prior to the model construction [17,18]. First, low variance features in the training set were removed using a threshold of 0.01. Second, if there existed two highly correlated features with a Pearson coefficient greater than 0.8, the feature with the highest mean absolute correlation to all other features within the training set was eliminated. Third, the least absolute shrinkage and selection operator (LASSO) with 10-fold cross-validation was performed on the training set to identify the most relevant subset of features for predicting breast cancer molecular subtypes. The final selected feature subset was then fixed and applied to the internal and external validation sets.

The categorical boosting (CatBoost) algorithm offering advantages in dealing with multi-class imbalanced data was employed to develop predictive models [19]. The MultiClassOneVsAll loss function was adopted, together with automatic class weighting (auto_class_weights = ‘SqrtBalanced’), to reduce the influence of class imbalance among breast cancer molecular subtypes during model training. After establishing the independent models, respectively, based on TIC-WIR and TIC-Area images, the output probabilities of these two models were calibrated using isotonic regression trained on the training set predictions and labels. The calibrated probabilities were then taken as input features to build the combined model and the learned calibration function was directly applied to the internal and external validation sets. In addition, an original magnetic resonance image-based (MR-ORI) model was also developed using the peak enhancement phase image of the tumor from the DCE-MRI sequence in order to compare with that built based on the information derived from TIC-based parametric images.

### 2.7. Statistical Analysis

**Figure 2:**
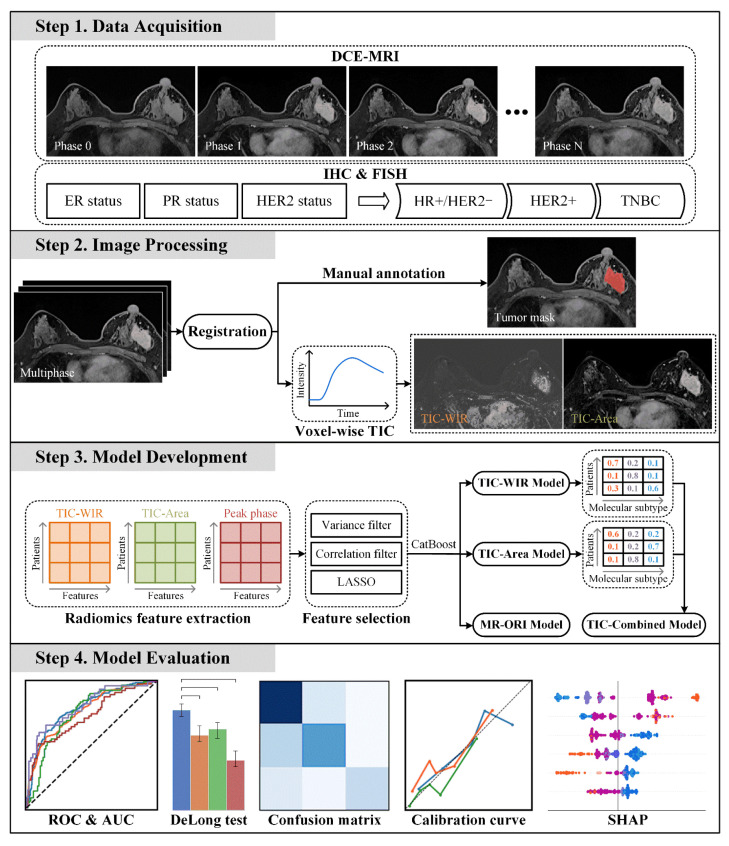
Overview of the study framework.

The discrimination of the different models was assessed through the receiver operating characteristic (ROC) curve and the area under the ROC curve (AUC), with pairwise AUC comparisons between the TIC-Combined model and the TIC-WIR model, TIC-Area model, and MR-ORI model performed using the DeLong test. The corresponding 95% confidence interval (CI) of the AUC was reported by resampling 1000 times via the bootstrap method. Other quantitative metrics including precision, recall, and F1-score were also calculated. The calibration curves were plotted to evaluate the agreement between predicted probabilities and actual outcomes. Additionally, the SHapley Additive exPlanations (SHAP) analysis was used to visualize the effect of each input feature on the prediction outcomes. All the analyses were performed in Python (version 3.8) and the overall workflow of the study is presented in Figure 2. A *p*-value less than 0.05 was considered statistically significant.

## 3. Results

### 3.1. Patient Characteristics

A total of 935 patients were ultimately included in this study. Of those, 845 patients from FUSCC were split into the training and internal validation sets using an 8:2 ratio by stratified sampling; 90 patients from RH were served as the external validation set. Table 1 summarizes the patient characteristics across all datasets.

### 3.2. Prediction Performance of Individual Models

Ultimately, five parametric radiomics features were, respectively, selected for the development of the TIC-WIR, TIC-Area, and MR-ORI models that established using heterogeneity information captured from TIC-based parametric images and original magnetic resonance images, as listed in Table 2. The ROC curves of these three models in the training, internal validation, and external validation sets are shown in Figure S1, combined with the micro-average and macro-average AUCs, as well as the one-vs-rest AUCs for each breast cancer molecular subtype. Compared to the MR-ORI model, the TIC-WIR and TIC-Area models demonstrated superior predictive performance with micro-average AUCs ranging from 0.69 to 0.74 and from 0.73 to 0.76, and macro-average AUCs ranging from 0.69 to 0.71 and from 0.69 to 0.73 across all datasets, respectively. In addition to the overall performance, the TIC-WIR and TIC-Area models’ discriminative ability for each molecular subtype of breast cancer outperformed that of the MR-ORI model. In the validation sets, the best subtype-specific AUC of 0.73 was achieved by the TIC-Area model for TNBC classification.

### 3.3. Prediction Performance of the Combined Model

**Figure 3:**
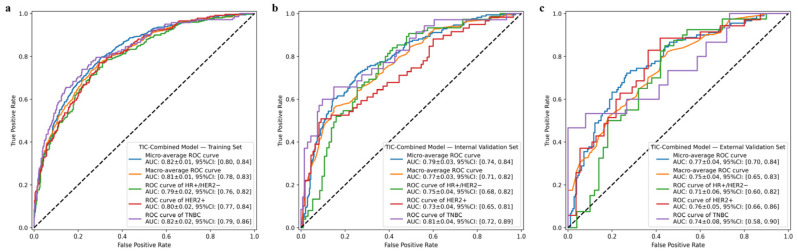
Receiver operating characteristic curves of the TIC-Combined model in the training (**a**), internal validation (**b**), and external validation (**c**) sets.

**Figure 4:**
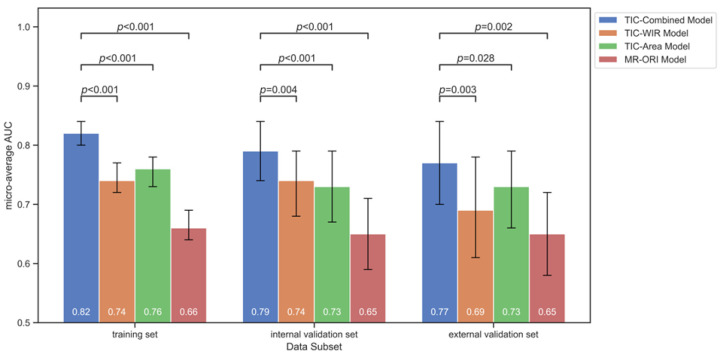
Pairwise comparison of micro-average AUCs between the established models in the training, internal validation, and external validation sets.

Based on the outputs of the TIC-WIR and TIC-Area models, a combined model was developed by leveraging the complementary strengths. The fused model combining information from both TIC-WIR and TIC-Area models (TIC-Combined) achieved higher AUCs compared to the individual models in both the overall and individual subtype prediction performance, as presented in Figure 3. For the micro-average AUC, the TIC-Combined model showed a statistically significant improvement over the TIC-WIR, TIC-Area, and MR-ORI models with values of 0.82 ± 0.01 (95% CI: 0.80–0.84), 0.79 ± 0.03 (95% CI: 0.74–0.84), and 0.77 ± 0.04 (95% CI: 0.70–0.84) in the training, internal validation, and external validation sets (all *p* < 0.05, Figure 4), respectively. For the one-vs-rest AUCs of each breast cancer molecular subtype, the TIC-Combined model outperformed the other three models in all datasets, although not all of the comparisons reached statistical significance (Figure S2). The TIC-Combined model achieved the highest subtype-specific AUC of 0.81 for TNBC prediction in the internal validation set and 0.76 for the HER2+ subtype prediction in the external validation set.

In addition, the confusion matrices and the other quantitative performance metrics of the TIC-Combined model across different datasets are, respectively, demonstrated in Figure S3 and Table S1. The calibration curves for HR+/HER2−, HER2+, and TNBC of the TIC-Combined model all indicated good concordance between predicted and observed breast cancer molecular subtypes in both validation sets (Figure S4).

### 3.4. Predictive Model Interpretability

**Figure 5:**
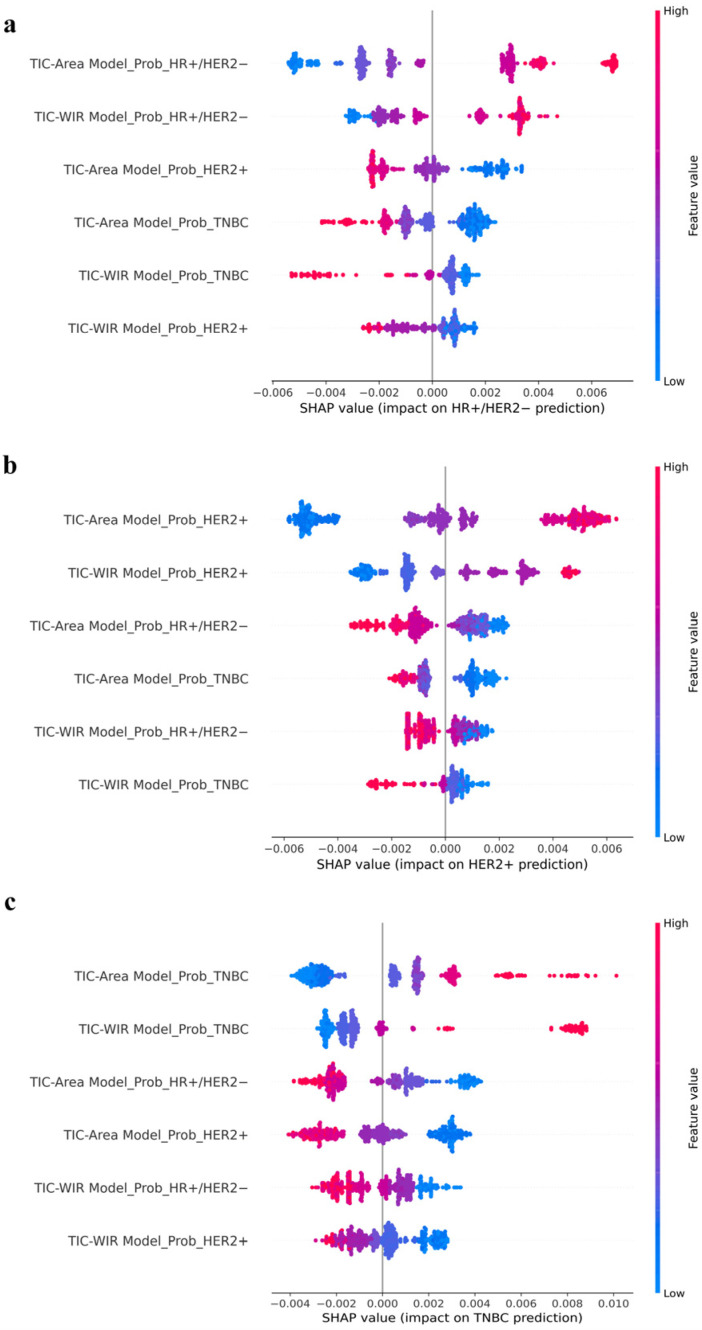
SHAP summary plots of the TIC-Combined model for predicting HR+/HER2− (**a**), HER2+ (**b**), and TNBC (**c**) subtypes in the training set.

The mean absolute SHAP values of input features from the TIC-WIR, TIC-Area, and MR-ORI models were analyzed to assess their relative importance in predicting each breast cancer molecular subtype, as summarized in Table 2. Features with higher SHAP values were considered more influential in the decision-making process of the model. The SHAP summary plots of the TIC-Combined model are presented in Figure 5, visualizing the contribution of individual features in identifying HR+/HER2−, HER2+, and TNBC subtypes. Across all breast cancer molecular subtypes, the most influential feature of the TIC-Combined model was the predicted probability from the TIC-Area model for the corresponding class, followed by that from the TIC-WIR model for the corresponding class.

## 4. Discussion

This study proposed voxel-wise TIC-based parametric images that leverages the rich spatiotemporal information of the whole tumor provided by DCE-MRI to non-invasively characterize contrast enhancement heterogeneity, aiming to improve the prediction of breast cancer molecular subtypes (HR+/HER2−, HER2+, and TNBC) and support individualized treatment strategies. The TIC-based parametric images were generated from two kinetic parameters, namely the wash-in rate indicating the speed of contrast uptake and the area under the TIC indicating the overall intensity of contrast uptake over the DCE-MRI examination time. We hypothesized that the heterogeneity among different breast cancer molecular subtypes could be reflected on TIC-based parametric images as differences in morphology, density, and texture characteristics, which were quantitatively captured through parametric radiomics analysis.

TIC-based parametric images provided superior predictive value for identifying breast cancer molecular subtypes compared to the original DCE-MRI images, as evidenced by the improved performance of the TIC-WIR and TIC-Area models over the MR-ORI model. Among the high-dimensional parametric radiomics features extracted, texture features accounted for the majority of the contributing variables in the predictive model construction. This finding aligns with recent studies suggesting texture heterogeneity helps differentiating the molecular subtypes in breast cancer more effectively than other categories of radiomic features [20,21,22]. Further, we examined the importance of original radiomics features within the TIC-WIR and TIC-Area models. Feature importance and SHAP analyses revealed that several texture features consistently contributed to subtype discrimination, supporting the biological relevance of the TIC-based radiomic representations. In addition, it is worth noting that the GLSZM feature of gray level nonuniformity normalized that extracted from wavelet-transformed images was valuable in both the TIC-WIR and TIC-Area models. Clinically, gray level nonuniformity normalized reflects the variability of gray-level intensity distributions within the tumor, which may correspond to heterogeneous microvascular perfusion, contrast agent leakage, and spatially varying permeability. Such enhancement heterogeneity is frequently observed in more aggressive breast cancer subtypes and may partially explain the discriminative power of this feature. By incorporating kinetic information across multiple phases and preserving voxel-level spatial details, TIC-based parametric images better reflect the spatiotemporal heterogeneous in tumor enhancement patterns than the single phase DCE-MRI image.

Notably, TIC-based parametric images that provide voxel-by-voxel kinetic information can be directly applied to the routine DCE-MRI protocols adopted in daily practice. Prior studies have also demonstrated pharmacokinetic parameters (Ktrans, Kep, Ve, Vp, and Slopemax) for their associations with histopathologic tumor characteristics and their potential utility in predicting treatment response [23,24]. However, the estimation of such physiologically meaningful quantitative parameters requires multiple critical factors including accurate arterial input function, complex mathematical modeling, and high temporal resolution data acquired over 5 to 6 min [25], which hinders its application in a clinical setting. In contrast, the TIC-WIR and TIC-Area kinetic parameters used in this study are derived from the routine DCE-MRI sequences that typically include only 4 to 5 post-contrast phases, making the proposed approach low-burden, protocol-compatible, and vendor-agnostic, and therefore readily integrable into standard clinical workflows. While the previous study has taken wash-out related parameters into consideration to explore possible TIC morphologies of breast lesions which relied on DCE-MRI images with 8 to 9 post-contrast phases [26], these parameters were not included in this study due to insufficient temporal coverage for capturing contrast agent clearance behavior during the delayed phase in routine clinical practice. Additionally, the robustness of the TIC-based models across both the internal and external validation sets provides further support for the broader clinical applicability of TIC-WIR and TIC-Area kinetic parameters.

Ultimately, the TIC-Combined model adopting a decision-level fusion strategy by integrating the calibrated probability outputs of the TIC-WIR and TIC-Area models showed the best performance for predicting breast cancer molecular subtypes. Rather than directly concatenating the respective input features extracted from TIC-WIR and TIC-Area images that may have the risk of feature redundancy and model overfitting, the fusion approach at the model output-level fully leverages the discriminative capabilities of each independent model. Moreover, all models in this study performed direct three-class classification for HR+/HER2−, HER2+, and TNBC subtypes instead of decomposing the task into multiple binary classifiers as done in most previous studies [27,28,29]. This approach enables the model to identify subtle differences across all three classes simultaneously and avoids potential conflicts in prediction results that can arise in one-vs-rest or one-vs-one schemes. From a clinical perspective, direct three-class modeling aligns more closely with real-world diagnostic processes and thereby enhances decision-making efficiency.

External validation further highlights the robustness of the TIC-Combined model. On the external dataset, precision, recall, and F1-scores were generally consistent with internal performance for HR+/HER2− and HER2+ subtypes, while TNBC exhibited lower recall of 0.53 and F1-score of 0.50, likely due to the smaller number of TNBC cases and higher heterogeneity in imaging features. The findings indicate that the TIC-Combined model, through feature normalization and the TIC-based approach, generalizes reasonably well across heterogeneous datasets. Clinically, this direct three-class modeling strategy aligns more closely with real-world diagnostic workflows and supports efficient, reliable decision-making across diverse patient populations.

Predictive model interpretability is also a central focus of this study for supporting clinical trust and decision-making. The SHAP summary plots of the TIC-Combined model revealed that the top two influential features for predicting each molecular subtype were the subtype-specific predicted probabilities from the TIC-Area and TIC-WIR models, with the TIC-Area output showing a more pronounced impact on the final prediction. This finding may be attributed to the fact that the TIC-Area kinetic parameter reflects the overall enhancement progress involving wash-in and wash-out behaviors over the entire DCE-MRI sequence, capturing a more comprehensive characterization of tumor perfusion compared to the TIC-WIR kinetic parameter.

Although immunohistochemistry remains the clinical gold standard for determining breast cancer molecular subtypes, the proposed imaging-based model is not intended to replace pathological assessment. Instead, it is designed to provide complementary, non-invasive information that may extend current clinical practice in several realistic scenarios. First, the model enables preoperative estimation of molecular subtype using routine DCE-MRI, offering early subtype-related insights before biopsy or surgical pathology results are available. This may support initial risk stratification and multidisciplinary decision-making at the time of diagnosis. Second, because IHC-based subtyping relies on tissue obtained from limited biopsy samples, it may not fully capture intratumoral heterogeneity. In contrast, our model analyzes the entire tumor volume and therefore has the potential to reflect spatial heterogeneity, which may be helpful for biopsy targeting or for interpreting discordant or equivocal pathological findings. Third, in selected clinical situations where biopsy is contraindicated, delayed, or yields insufficient tissue, the imaging-based subtype prediction may serve as a supplementary reference for treatment planning and clinical monitoring. Finally, the proposed approach may also be valuable in research settings for non-invasive patient stratification and imaging-phenotype association studies, particularly in large retrospective cohorts. Taken together, these potential applications highlight the role of the proposed model as an adjunct to standard IHC-based subtyping, providing whole-tumor characterization that may enhance clinical confidence and decision support rather than replacing established diagnostic pathways.

Some limitations of our study should be noted. First, assessments of radiomics feature stability (such as intra- or inter-observer variability, sensitivity to segmentation perturbations, resampling variations, or discretization parameters) were not performed. In our workflow, tumor segmentations were manually delineated by one experienced radiologist and subsequently reviewed by a second senior radiologist to ensure accuracy. This dual-review procedure helps minimize segmentation errors, and radiomics features are generally robust to minor variations in segmentation. Nevertheless, the lack of stability testing represents a limitation. Second, additional molecular correlates, such as Ki67, were not included in this study. As a result, our analysis focuses on the three clinically commonly used breast cancer subtypes (HR+/HER2−, HER2+, and TNBC) and does not further distinguish Luminal A and Luminal B subtypes. Third, the retrospective design of this study may introduce potential selection bias. Although the model was validated using an external dataset from another medical center, the external cohort is relatively small, particularly for the TNBC subgroup. These limitations may affect the generalizability and could influence subtype-specific performance due to class imbalance, highlighting the need for prospective large-sample validation across multiple centers. Fourth, breast tumors were manually annotated by experienced radiologists that is time-consuming. In future work, breast tumors can be initially segmented using automated algorithms and subsequently reviewed by radiologists to improve efficiency. Fifth, this study performed interpretability analysis using SHAP to quantify the contribution of each feature to the model predictions. However, the underlying biological significance of these features were not further explored because the current dataset did not contain matched molecular or pathological data. Sixth, the MR-ORI baseline model was limited to single-phase radiomics. Future studies incorporating multiphase radiomics or semi-quantitative kinetic maps may provide a more comprehensive comparison. Seventh, the sensitivity of TIC-derived parameters to variations in temporal resolution and contrast arrival time was not assessed. Although voxel-wise normalization and relative enhancement patterns help mitigate these effects, future studies are warranted to systematically evaluate the robustness of TIC-based radiomics features across different imaging protocols and temporal resolutions.

## 5. Conclusions

In conclusion, parametric radiomics from TIC-based parametric images enables effectively HR+/HER2−, HER2+, and TNBC subtype prediction, demonstrating potential to serve as non-invasive imaging tool indicative of tumor enhancement heterogeneity in different breast cancer molecular subtypes.

## Figures and Tables

**Table 1:** Patient characteristics and DCE-MRI parameters in each dataset.

Characteristic	Training Set	Internal Validation Set	External Validation Set
Number of Patients	676	169	90
Age (years)	52 (44–59)	51 (43–58)	56 (46–65)
Tumor Maximal Diameter (cm)	5.24 (3.77–7.64)	5.51 (3.43–7.44)	3.11 (2.22–4.40)
Molecular Subtype of Breast Cancer			
HR+/HER2−	299	75	40
HER2+	235	59	35
TNBC	142	35	15
Equipment	Siemens	Siemens	Philips
Number of Post-contrast Phases	5	5	4
Repetition Time (ms)	4.5	4.5	4.3
Echo Time (ms)	1.6	1.6	2.1
Flip Angle (°)	10	10	12
In-plane Resolution (mm × mm)	0.44 × 0.44–1.31 × 1.31	0.44 × 0.44–0.94 × 0.94	0.59 × 0.59–0.81 × 0.81
Slice Thickness (mm)	1.3–2.2	1.3–2.2	1.0–2.6

Age and tumor maximal diameter are presented as the median with the 25th and 75th percentiles in parentheses. DCE-MRI, dynamic contrast-enhanced magnetic resonance imaging; HER2, human epidermal growth factor receptor 2; HR, hormone receptor; TNBC, triple-negative breast cancer.

**Table 2:** Mean absolute SHAP values of each feature in the TIC-WIR, TIC-Area, and MR-ORI models for the prediction of HR+/HER2−, HER2+, and TNBC subtypes.

Model	Feature Name	mSHAP (HR+/HER2−)	mSHAP (HER2+)	mSHAP (TNBC)
TIC-WIR Model	original_glcm_SumEntropy	0.0823	0.0594	0.1235
	wavelet-LLH_glszm_GrayLevelNonUniformityNormalized	0.1587	0.0548	0.2076
	wavelet-HHL_firstorder_Skewness	0.0890	0.1673	0.1006
	wavelet-HHL_glszm_HighGrayLevelZoneEmphasis	0.0951	0.0867	0.1330
	wavelet-LLL_ngtdm_Busyness	0.1187	0.0219	0.1352
TIC-Area Model	original_shape_Elongation	0.0649	0.1683	0.2085
	wavelet-LLH_glszm_GrayLevelNonUniformityNormalized	0.1944	0.0946	0.1656
	wavelet-LHL_glszm_SmallAreaEmphasis	0.1235	0.0497	0.1724
	wavelet-HLL_gldm_LargeDependenceLowGrayLevelEmphasis	0.0584	0.1232	0.2044
	wavelet-HLH_firstorder_TotalEnergy	0.1407	0.1460	0.1159
MR-ORI Model	wavelet-LHL_gldm_LowGrayLevelEmphasis	0.0048	0.0033	0.0061
	wavelet-HLH_gldm_LargeDependenceLowGrayLevelEmphasis	0.0044	0.0023	0.0072
	wavelet-HLH_glszm_LowGrayLevelZoneEmphasis	0.0017	0.0005	0.0007
	wavelet-LLL_gldm_DependenceVariance	0.0055	0.0077	0.0103
	wavelet-LLL_glszm_SmallAreaEmphasis	0.0042	0.0007	0.0029

SHAP, SHapley Additive exPlanations; mSHAP, mean absolute SHAP value; TIC, time-intensity curve; TIC-WIR, wash-in rate derived from the TIC; TIC-Area, area under the TIC; MR-ORI, original magnetic resonance images; HER2, human epidermal growth factor receptor 2; HR, hormone receptor; TNBC, triple-negative breast cancer.

## Data Availability

The data presented in this study are available on request from the corresponding authors due to patient confidentiality.

## Supplementary Materials

The following supporting information can be downloaded at: https://www.mdpi.com/article/10.3390/tomography12020027/s1, Figure S1: Receiver operating characteristic curves of the MR-ORI (a–c), TIC-WIR (d–f), and TIC-Area (g–i) models in the training, internal validation, and external validation sets; Figure S2: Pairwise comparison of AUCs for HR+/HER2− (a), HER2+ (b), and TNBC (c) subtypes between the established models in the training, internal validation, and external validation sets; Figure S3: Confusion matrices of the TIC-Combined model for predicting breast cancer molecular subtypes in the training (a), internal validation (b), and external validation (c) sets; Figure S4: Calibration curves of the TIC-Combined model for HR+/HER2−, HER2+, and TNBC subtype prediction in the validation sets; Table S1: Classification performance of the TIC-Combined model in terms of precision, recall, and F1-score for each breast cancer molecular subtype prediction, along with overall metrics including the micro-average, macro-average, and weighted-average.

## Abbreviations

ER	Estrogen receptor
PR	Progesterone receptor
HR	Hormone receptor
HER2	Human epithelial growth factor receptor 2
IHC	Immunohistochemistry
TNBC	Triple-negative breast cancer
TIC	Time-intensity curve
WIR	Wash-in rate
GLCM	Gray level co-occurrence matrix
GLSZM	Gray level size zone matrix
GLRLM	Gray level run length matrix
NGTDM	Neighboring gray tone difference matrix
GLDM	Gray level dependence matrix
LASSO	Least absolute shrinkage and selection operator
CatBoost	Categorical boosting
MR-ORI	Original magnetic resonance image-based
ROC	Receiver operating characteristic
AUC	Area under the curve
CI	Confidence interval
SHAP	SHapley Additive exPlanations
