# Supplementary material for: Dynamic Contrast-Enhanced MRI Kinetic Curve-Driven Parametric Radiomics for Predicting Breast Cancer Molecular Subtypes: A Multicenter and Interpretable Study
Source: Tomography. 2026 Feb 22;12(2):27. doi: 10.3390/tomography12020027 (PMC12944185; doi:10.3390/tomography12020027)
Supplement: Supplementary file 1 [file tomography-12-00027-s001.zip › tomography-4118118-supplementary.pdf]

Table S1. Classification performance of the TIC-Combined model in terms of precision, recall, and F1-score for each breast cancer molecular subtype prediction, along with overall metrics including the micro-average, macro-average, and weighted-average.

Classes	Training Set			Internal Validation Set			External Validation Set		
	Precision	Recall	F1-score	Precision	Recall	F1-score	Precision	Recall	F1-score
HR+/HER2−	0.65	0.76	0.70	0.63	0.83	0.71	0.59	0.75	0.66
HER2+	0.64	0.58	0.61	0.68	0.51	0.58	0.77	0.49	0.60
TNBC	0.62	0.49	0.55	0.65	0.49	0.56	0.47	0.53	0.50
Micro Average	0.64	0.64	0.64	0.64	0.64	0.64	0.61	0.61	0.61
Macro Average	0.64	0.61	0.62	0.65	0.61	0.62	0.61	0.59	0.59
Weighted Average	0.64	0.64	0.64	0.65	0.64	0.64	0.64	0.61	0.61

Abbreviations: HER2, human epidermal growth factor receptor 2; HR, hormone receptor; TNBC, triple-negative breast cancer; TIC, time-intensity curve; TIC-Combined, combination of wash-in rate derived from the TIC and the area under the TIC.

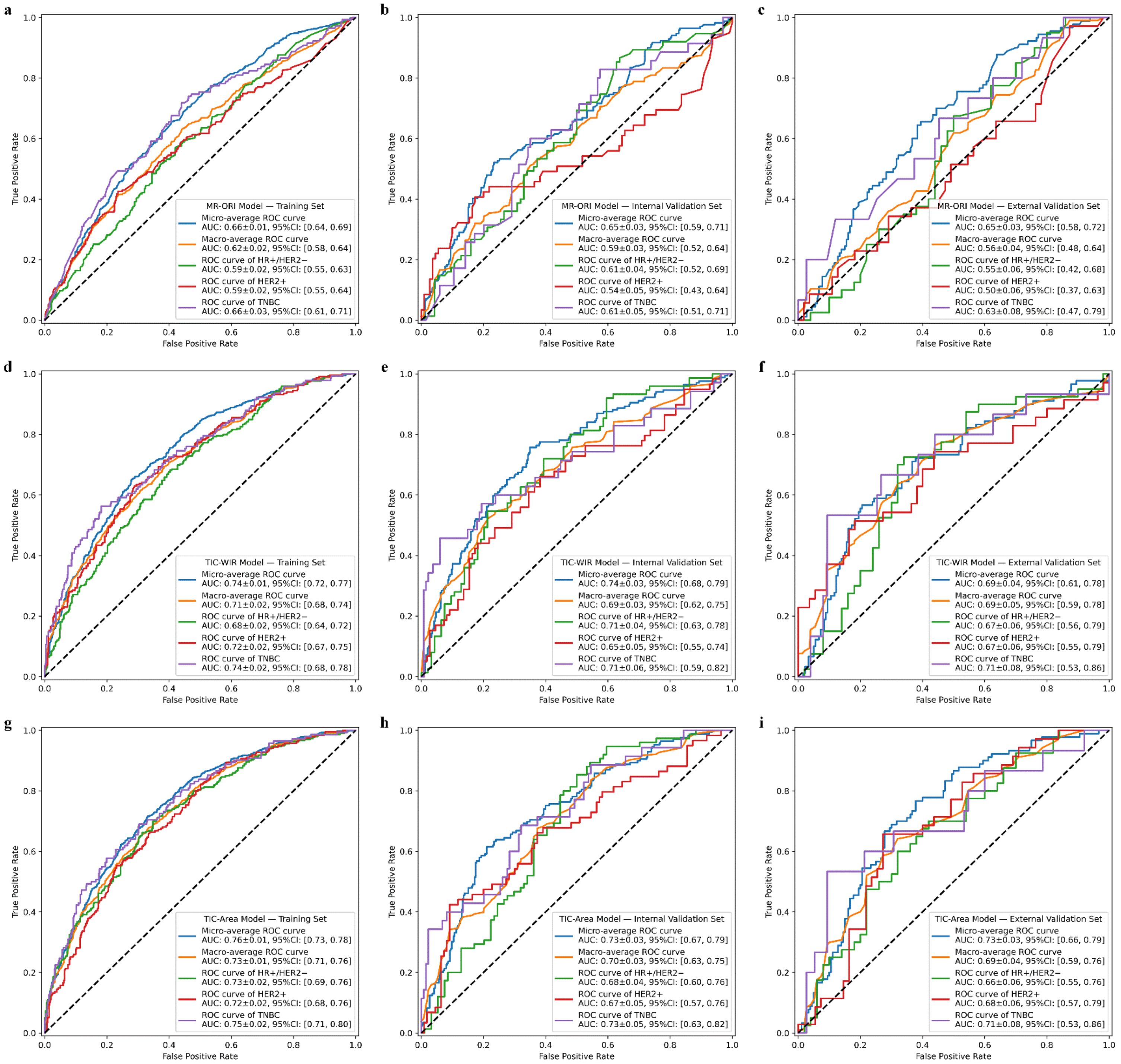

Figure S1. Receiver operating characteristic curves of the MR-ORI (**a-c**), TIC-WIR (**d-f**), and TIC-Area (**g-i**) models in the training, internal validation, and external validation sets. ROC, receiver operating characteristic curve; AUC, area under the ROC curve; CI, confidence interval; MR-ORI, original magnetic resonance images; TIC, time-intensity curve; TIC-WIR, wash-in rate derived from the TIC; TIC-Area, area under the TIC; HER2, human epidermal growth factor receptor 2; HR, hormone receptor; TNBC, triple-negative breast cancer.

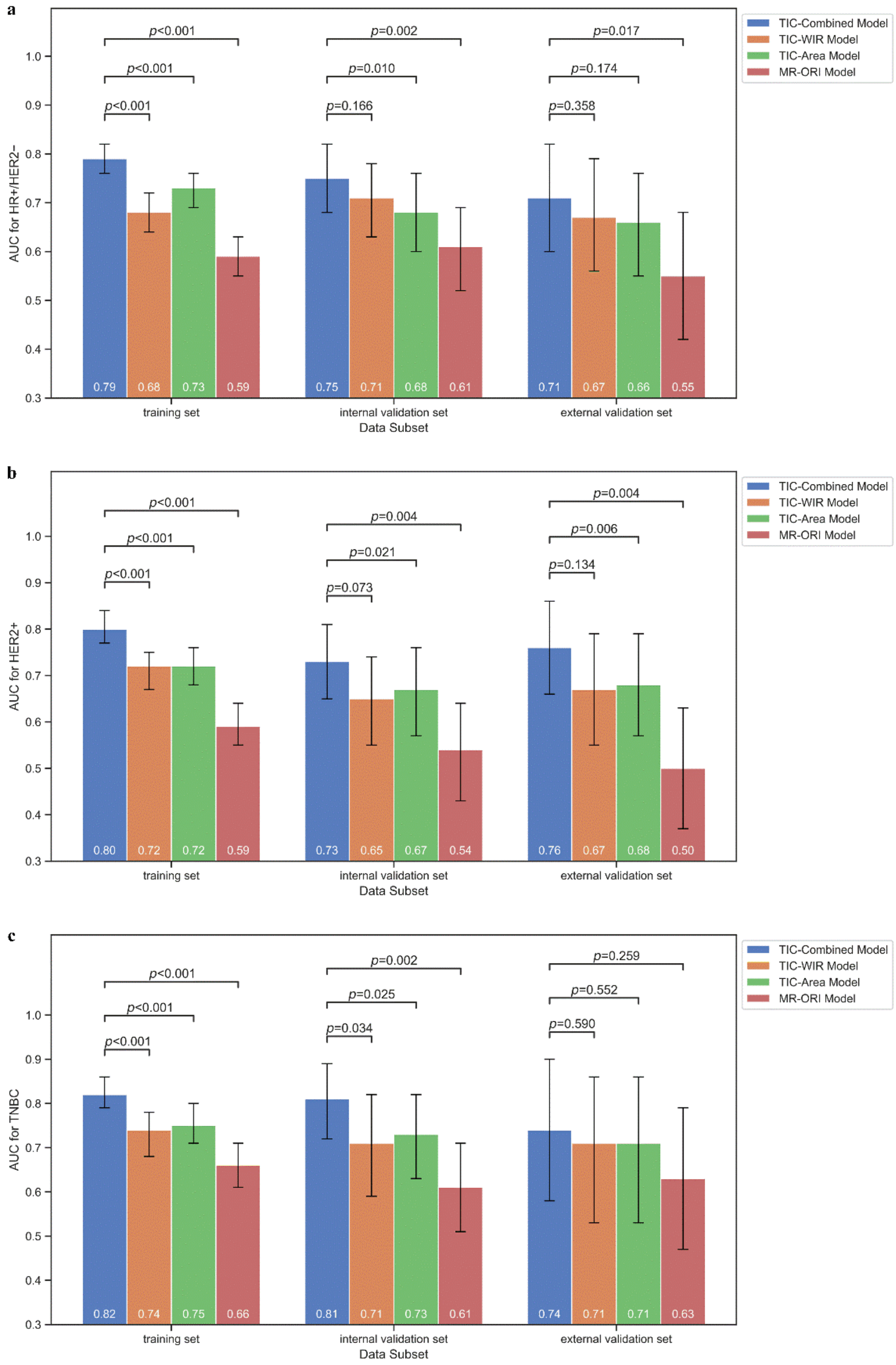

Figure S2. Pairwise comparison of AUCs for HR+/HER2− (**a**), HER2+ (**b**), and TNBC (**c**) subtypes between the established models in the training, internal validation, and external validation sets. AUC, area under the receiver operating characteristic curve; TIC, time-intensity curve; TIC-WIR, wash-in rate derived from the TIC; TIC-Area, area under the TIC; TIC-Combined, combination of TIC-WIR and TIC-Area; MR-ORI, original magnetic resonance images. HER2, human epidermal growth factor receptor 2; HR, hormone receptor; TNBC, triple-negative breast cancer.

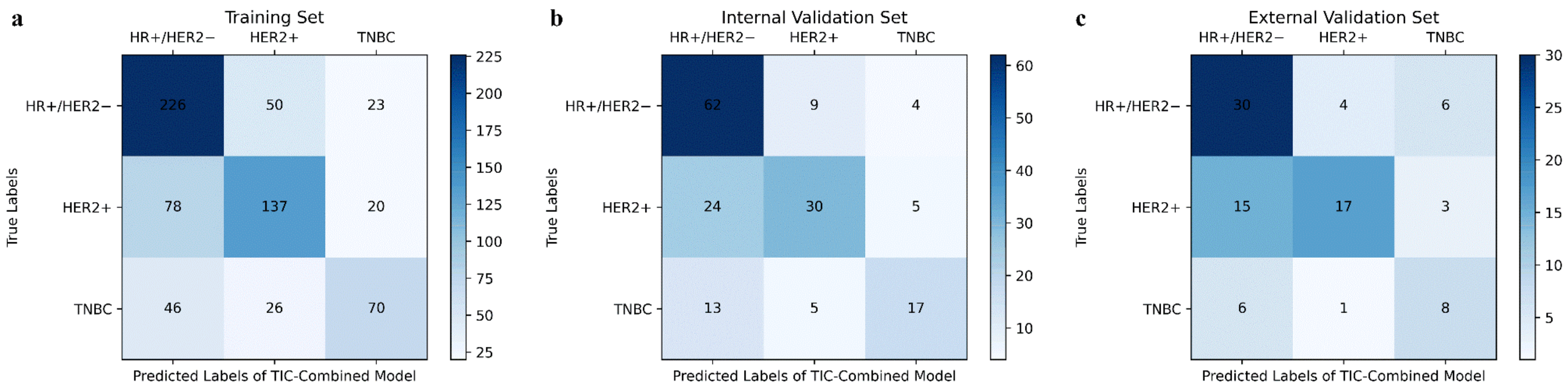

Figure S3. Confusion matrices of the TIC-Combined model for predicting breast cancer molecular subtypes in the training (**a**), internal validation (**b**), and external validation (**c**) sets. HER2, human epidermal growth factor receptor 2; HR, hormone receptor; TNBC, triple-negative breast cancer; TIC, time-intensity curve; TIC-Combined, combination of wash-in rate derived from the TIC and the area under the TIC.

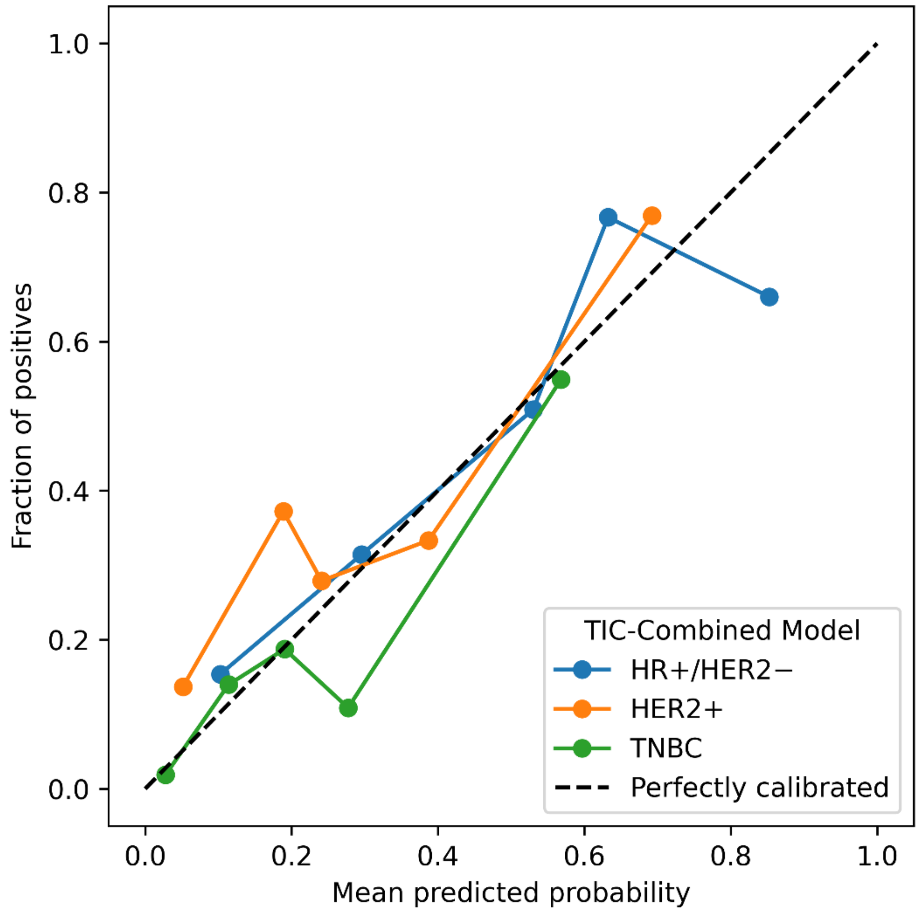

Figure S4. Calibration curves of the TIC-Combined model for HR+/HER2−, HER2+, and TNBC subtype prediction in the validation sets. HER2, human epidermal growth factor receptor 2; HR, hormone receptor; TNBC, triple-negative breast cancer; TIC, time-intensity curve; TIC-Combined, combination of wash-in rate derived from the TIC and the area under the TIC.
